# Novel de novo *EEF1A2* missense mutations causing epilepsy and intellectual disability

**DOI:** 10.1002/mgg3.219

**Published:** 2016-04-03

**Authors:** Wayne W.K. Lam, John J. Millichap, Dinesh C. Soares, Richard Chin, Ailsa McLellan, David R. FitzPatrick, Frances Elmslie, Melissa M. Lees, G. Bradley Schaefer, DDD study, Catherine M. Abbott

**Affiliations:** ^1^ South East of Scotland Clinical Genetics ServiceCrewe RoadEdinburghUK; ^2^ Centre for Genomic & Experimental MedicineMRC Institute of Genetics and Molecular MedicineUniversity of EdinburghWestern General HospitalCrewe RoadEdinburghEH4 2XUUK; ^3^ Muir Maxwell Epilepsy CentreUniversity of Edinburgh20 Sylvan PlaceEdinburghEH9 1UWUK; ^4^ Paediatric NeurosciencesRoyal Hospital for Sick ChildrenSciennes RoadEdinburghEH9 1LFUK; ^5^ Epilepsy CenterDepartments of Pediatrics and NeurologyAnn & Robert H. Lurie Children's Hospital of ChicagoNorthwestern University Feinberg School of Medicine225 E Chicago AveBox #29ChicagoIllinois60611; ^6^ MRC Human Genetics UnitMRC Institute of Genetics and Molecular MedicineUniversity of EdinburghWestern General HospitalCrewe RoadEdinburghEH4 2XUUK; ^7^ Child Life and HealthUniversity of Edinburgh20 Sylvan PlaceEdinburghEH9 1UWUK; ^8^ South West Thames Regional Genetics ServiceSt George's HospitalTootingLondonUK; ^9^ Department of Clinical GeneticsGreat Ormond Street HospitalGreat Ormond StreetLondonUK; ^10^ Division of Medical GeneticsArkansas Children's HospitalLittle RockArkansas; ^11^ DDD StudyWellcome Trust Sanger InstituteHinxtonCambridgeUK

**Keywords:** Autism, *EEF1A2*, epilepsy, intellectual disability, translation elongation

## Abstract

**Background:**

Exome sequencing has led to the discovery of mutations in novel causative genes for epilepsy. One such gene is *EEF1A2*, encoding a neuromuscular specific translation elongation factor, which has been found to be mutated de novo in five cases of severe epilepsy. We now report on a further seven cases, each with a different mutation, of which five are newly described.

**Methods:**

New cases were identified and sequenced through the Deciphering Developmental Disabilities project, via direct contact with neurologists or geneticists, or recruited via our website.

**Results:**

All the mutations cause epilepsy and intellectual disability, but with a much wider range of severity than previously identified. All new cases share specific subtle facial dysmorphic features. Each mutation occurs at an evolutionarily highly conserved amino acid position indicating strong structural or functional selective pressure.

**Conclusions:**

*EEF1A2* should be considered as a causative gene not only in cases of epileptic encephalopathy but also in children with less severe epilepsy and intellectual disability. The emergence of a possible discernible phenotype, a broad nasal bridge, tented upper lip, everted lower lip and downturned corners of the mouth may help in identifying patients with mutations in *EEF1A2*.

## Introduction

The advent of family based exome sequencing has facilitated the discovery of de novo and/or inherited ultrarare mutations in an increasingly large number of genes as the cause of epilepsy. Some of these genes have quite unexpected cellular functions. In these cases, the burden of proof that the mutations are causative rests not just on the fact that they have occurred de novo*,* and the absence of such mutations in unaffected individuals, but also on the predicted effects on the protein, and whether more than one mutation is found in the same gene (or the same mutation is found in multiple affected individuals).

One gene in which missense mutations have recently been reported is *EEF1A2*, encoding translation elongation factor 1A2 (OMIM #602959). Elongation factor eEF1A has a key role in protein synthesis, the delivery of amino‐acylated tRNAs to the ribosome. In addition to this role, eEF1A has been reported to have numerous noncanonical “moonlighting” properties, possibly by virtue of its abundance in the cell, where it comprises 3% of the total protein (Condeelis 1995).

While mutations affecting such a basic housekeeping function would at first glance seem unlikely to affect only neurodevelopment, the specificity of the disorder is determined by the expression pattern of eEF1A isoforms. All vertebrates have two independently encoded isoforms of eEF1A; eEF1A1 is ubiquitously expressed throughout development but is then switched off specifically in neurons and muscle postnatally, where it is replaced by eEF1A2 (Chambers et al. 1998; Khalyfa et al. 2003). eEF1A2 is unique among translation factors in being tissue‐specific. It is expressed only in neurons and muscle, with minor specific sites of expression in pancreatic islet cells and enteroendocrine cells. Complete loss of function of eEF1A2 in the mouse (via a homozygous deletion of the promoter and first exon) causes severe neurodegeneration, loss of muscle bulk and death by 4 weeks (Chambers et al. 1998). Mice that are heterozygous for this loss of function mutation, on the other hand, have no gross motor problems even at 21 months (Griffiths et al. 2012).

In humans, three distinct missense mutations in *EEF1A2* have been reported in five patients. The initial report was in a study of individuals with severe intellectual disability (ID): a Gly70Ser mutation was found in a female with severe ID, autistic features and aggressive behaviors. She was also reported to have myoclonic, absence, and grand mal seizures (de Ligt et al. 2012). The second report was in a survey of children with epileptic encephalopathy, where the same Gly70Ser mutation was identified in a 14‐year‐old boy with refractory epilepsy. He also has limited comprehension and is nonverbal (Veeramah et al. 2013). A third Gly70Ser mutation was reported in a 1‐year‐old girl, but no clinical information was included beyond a broad categorization as “neurological with other systems involvement” (Yang et al. 2014). The fourth and fifth individuals were identified in Japan; both have autism and severe ID. One had infantile spasms, now controlled by valproate, the other has generalized tonic seizures. In addition, both girls were said to have characteristic facial features. They each have different missense mutations, Glu122Lys and Asp252His, respectively (Nakajima et al. 2015). Two further cases of Glu122Lys mutations have recently been described (Inui et al. 2015).

We now report the finding of further mutations, all of which have arisen de novo on one allele in the affected individuals. We report on seven cases, each with a different mutation, of which five are newly described. All are associated with epilepsy and ID, but with a much wider range of severity than previously suspected. We note characteristic facial features in all patients for whom we could obtain photos.

## Methods

### Ethical compliance

This study has UK Research Ethics Committee approval (10/H0305/83, granted by the Cambridge South REC, and GEN/284/12 granted by the Republic of Ireland REC).

### Recruitment

Patients were recruited through personal communication with clinicians, via a website (http://eef1a2epilepsy.wordpress.com/) and from the Deciphering Developmental Disorders (DDD) project (http://www.ddduk.org/). A *pro forma* was designed and sent to clinicians; where possible this was used in conjunction with the information deposited in DDD.

### Sequencing and analysis

Trio‐based exome sequencing was performed as part of the DDD study as previously described (Deciphering Developmental Disorders 2015; Wright et al. 2015). In brief, target capture using Agilent SureSelect 55 MB Exome Plus was performed on saliva‐ or blood‐derived genomic DNA from each affected individual and their parents and sequenced on Illumina HiSeq. DeNovoGear21 was used to identify de novo sequence variants and Ensembl Variant Effect Predictor (VEP version 2.6, http://www.ensembl.org/info/docs/tools/vep/index.html) was used to predict the effect of each genomic variant. PolyPhen‐2 analysis was carried out at http://genetics.bwh.harvard.edu/pph2/. SIFT analysis was carried out at http://sift.bii.a-star.edu.sg/www/SIFT_seq_submit2.html (parameters used: Database UniProt‐SwissProt2010_09; Median conservation of sequences 3.00; Remove sequences more than 90% identical). Genbank accession for *EEF1A2* is NM_001958.3.

## Results

### Clinical findings/case studies

#### Case 1

Case 1 is a 3‐year‐old girl born after a normal pregnancy at 38 weeks, weighing 3.72 kg (75th centile). She developed seizures at age 2 months and was diagnosed with an epileptic encephalopathy. Initially she had very frequent myoclonic and tonic‐clonic seizures and later developed absence seizures. Her EEG showed multifocal and occasional generalized discharges. After starting sodium valproate at age 2 years, her seizure frequency has decreased to 1–2 per day. She has global developmental delay, hypotonia and dysphagia. She is unable to walk. She has a small hemangioma on her left temple, for which there is a family history on the mother's side, and her head circumference was 46.5 cm at 9 months (91st centile) but no particular dysmorphisms were noted. She was found to have a heterozygous constitutive c.208G‐>A (p.G70S) mutation in *EEF1A2* that was not detected in either parent.

#### Case 2

Case 2 is a 9‐year‐old boy born by emergency Cesarean section at 42 weeks after an uncomplicated pregnancy, weighing 4.45 kg (98th centile). His mother has a history of epilepsy but was not taking anticonvulsants during pregnancy. He was described as a passive baby and now has severe global developmental delay and seizures. He did not sit independently until 13 months or walk until over 5 years. He does not speak recognizable words but uses signs to communicate his needs. He was reported to have brachycephaly, widely spaced teeth, an everted lower lip and head circumference of 52 cm at 5 years (>25th centile). He was found to have a heterozygous constitutive c.211A‐>C (p.I71L) mutation in *EEF1A2* not detected in either parent.

#### Case 3

Case 3 is a 14‐year‐old girl born after a normal pregnancy at 37 weeks, weighing 2.81 kg (25th centile). There was initial poor weight gain and she had three hospital admissions in the first year with pulmonary infections. Developmental delay was identified from 8 months, with delay in motor milestones; she only sat from 14 months. Seizure onset was at age 2 years, sometime after her developmental delay was first noted. The seizures were characterized by head drops which then evolved to eye rolling and arm extension occurring in clusters reminiscent of infantile spasms. She has continued to have seizures which have been resistant to medication. Head circumference measured at age 8.75 years was 51.5 cm (<50th centile). She is still unable to walk independently and has no speech. She has brachycephaly with small wide‐spaced teeth. She is generally hypotonic and has cold peripheries. She now has reduced bone density with fractures and thoracolumbar scoliosis. She was found to have a heterozygous de novo constitutive c.271G‐>A (p.D91N) mutation in *EEF1A2*.

#### Case 4

Case 4 is a 9‐year‐old girl who was born weighing 3.86 kg (>75th centile). She had focal seizures as an infant but now has myoclonic, tonic, and occasional tonic‐clonic seizures, occurring daily. She has severe hypotonia and severe global delays. She has no head control, no ability to grasp objects, is nonverbal and unable to sit or bear weight on legs. She has very poor bone density. Her head circumference was 43.6 cm at 6 months (<75th centile), and 50 cm at 6 years (>2nd). She was found to have a heterozygous de novo c.292T‐>C (p.F98L) mutation in *EEF1A2*.

#### Case 5

Case 5 is a 6‐year‐old girl born after a normal pregnancy at 41 weeks, weighing 3.43 kg (50th centile). Hypotonia was noted during the neonatal period with subsequent gross motor delay. She sat unsupported at 15 months and walked independently from 4 years. She has no speech but vocalizes and uses signs. Seizures developed at 10 weeks as jerky, stiffening of legs several times a week, which then developed to include head nodding. Her symptoms improved on commencement of sodium valproate. An initial EEG at 10 months was normal but showed spikes and polyspikes with some slow wave activity at 21 months. She was described to have downslanting and slightly short palpebral fissures, a broad forehead and a broad, flat nasal bridge with downturned corners of the mouth and head circumference below the 2nd centile. She was found to have a heterozygous de novo constitutive c364G‐>A (p.E122K) mutation in *EEF1A2*.

#### Case 6

Ten‐year‐old girl born at 40 weeks by planned Cesarean section weighing 3.69 kg (<75th centile). Initial gross and fine motor development was normal but only walked independently at 2 years. No evidence of hypotonia. Speaks in sentences, with significant delays in language and comprehension but pleasant and friendly; in mainstream primary but will go to special school at secondary level. Gait and coordination immature but essentially normal. Seizures commenced at 3 months, and were initially myoclonic seizures. She then developed absence seizures at the age of 2 years which are now controlled by valproate and lamotrigine. EEG shows slow background rhythms and generalized epileptic discharges. Her MRI brain is normal. Head circumference 51 cm at 5.2 years (25th centile). Broad base nose, prominent midface and thin upper lip but normal eyes. Found to have heterozygous de novo constitutive c.370G‐>A (p.E124K) mutation in *EEF1A2*.

#### Case 7

Case 7 is a 5‐year‐old boy born at 41 weeks by Cesarean section for decreased fetal heart rate weighing 2.80 kg (9th centile) following an uncomplicated pregnancy. Family history negative for seizures or developmental concerns in either parent, but the boy has a maternal uncle with developmental delays who is able to live independently. Developmental history notable for sitting with support at 18 months and currently he does not walk, has no speech, and is able to help feed himself. Past medical history significant for food allergies, eosinophilic esophagitis, and pancreatitis. He is frequently irritable and uncomfortable – he cries and grinds his teeth, kicking his legs and flapping his hands. He has choreic movements and disturbed sleep which responds partially to melatonin. His first seizure was at 4 months and was characterized by rightward eye deviation and right limb motor activity. Initial EEG was abnormal and brain MRI abnormal due to mild hypoplasia of corpus callosum and mild global volume loss (that is progressive on subsequent MR neuroimaging). He started phenobarbital and had no further seizures until 6 months old when he developed infantile spasms. Despite multiple rounds of ACTH/steroids and multiple anticonvulsant trials he continues to have seizures of multiple types including isolated epileptic spasms, myoclonic, myoclonic‐tonic, tonic, and tonic‐clonic seizures. His head circumference was 50 cm at 5 years (>25th centile). This boy was found to have a heterozygous de novo c.1267 C>T (p.R423C) mutation in *EEF1A2*. The clinical findings are summarized, together with those in previous reports, in Table 1.

**Table 1:** Summary of phenotypic characteristics seen in each case with a missense mutation in *EEF1A2*

Protein change	Epilepsy	Autism	SID/delay/motor delay	Hypotonia	Facial features noted	Other	Reference
Gly70Ser	Myoclonic seizures, grand mal, absences	Yes	Global developmental delay, SID	Yes	No	Normal head circumference at 22 years	de Ligt et al. 2012
Gly70Ser	Infantile spasms, then myoclonic, refractory	NR	Non‐verbal	Yes	NR	Acquired microcephaly <2nd centile, gait instability	Veeramah et al. 2013
Gly70Ser	Epileptic encephalopathy	NR	Global developmental delay	Yes	Yes	No microcephaly at 9 months, dysphagia	Yang et al. 2014, a This study, case 1
Ile71Leu	Seizures	NR	Global developmental delay	NR	Yes	Head circumference >9th centile, brachycephaly	This study, case 2
Asp91Asn	Head drops, then infantile spasms. Continued head drops and atypical absences	NR	Global developmental delay. Non‐verbal	Yes	Yes	Head circumference <50th centile. Brachycephaly.Reduced bone density with fractures	This study, case 3
Phe98Leu	Focal seizures as infant, now myoclonic, tonic and occasional tonic‐clonic	No	Global developmental delay	Yes	Yes	Head circumference 85th centile at 6 months, Reduced bone density.	This study, case 4
Glu122Lys	Infantile spasms, controlled	No	Non‐verbal, gross motor delay	Yes	Yes	Head circumference <2nd centile at 5 years, unsteady gait	This study, case 5
Glu122Lys	Infantile spasms, controlled	Yes	Yes	Yes	Yes	Small head circumference at birth, cerebral atrophy, ataxic gait	Nakajima et al. 2015
Glu124Lys	Myoclonic seizures from 3 months Absences from 2 years, controlled	No	Significant delays in language but speaks in sentences	No	No	Head circumference 25th centile at 5 years, gait immature but essentially normal	This study, case 6
Asp252His	Generalized tonic seizures, controlled	Yes	Yes	Yes	Yes	Small head circumference at birth, cerebral atrophy	Nakajima et al. 2015
Arg423Cys	Multiple daily seizures, intractable	NR	Global developmental delay	Yes	Yes	Head circumference 25th centile at 4 years, multiple food allergies	This study, case 7

a Categorised under “Patients with Neurologic Plus Other Organ System Disease Phenotype” in Yang et al.

All the mutations are missense, and have arisen de novo. Each of the individuals identified as having a mutation in *EEF1A2* has experienced repeated seizures, and in some cases they now have intractable epilepsy. Almost all show global developmental delay and hypotonia, but one individual, case 6, is clearly much less severely affected than the others. She has much milder but still moderate ID, and has had no seizures for a number of years.

In contrast to the previously published cases, none of the individuals we report has been diagnosed with autism. Head circumference is frequently, but not invariably, affected, but in the absence of longitudinal data it is hard to know whether this could be associated with specific mutations, or whether it varies with age. Gait abnormalities have been reported in the two individuals with E122K mutations, and one of the children with a G70S mutation. It is impossible to tell whether this would be consistently associated with mutations in *EEF1A2*, or only with specific mutations, as many of the patients are unable to walk. Other individuals have previously undescribed features like food allergies and reduced bone density.

**Figure 1:**
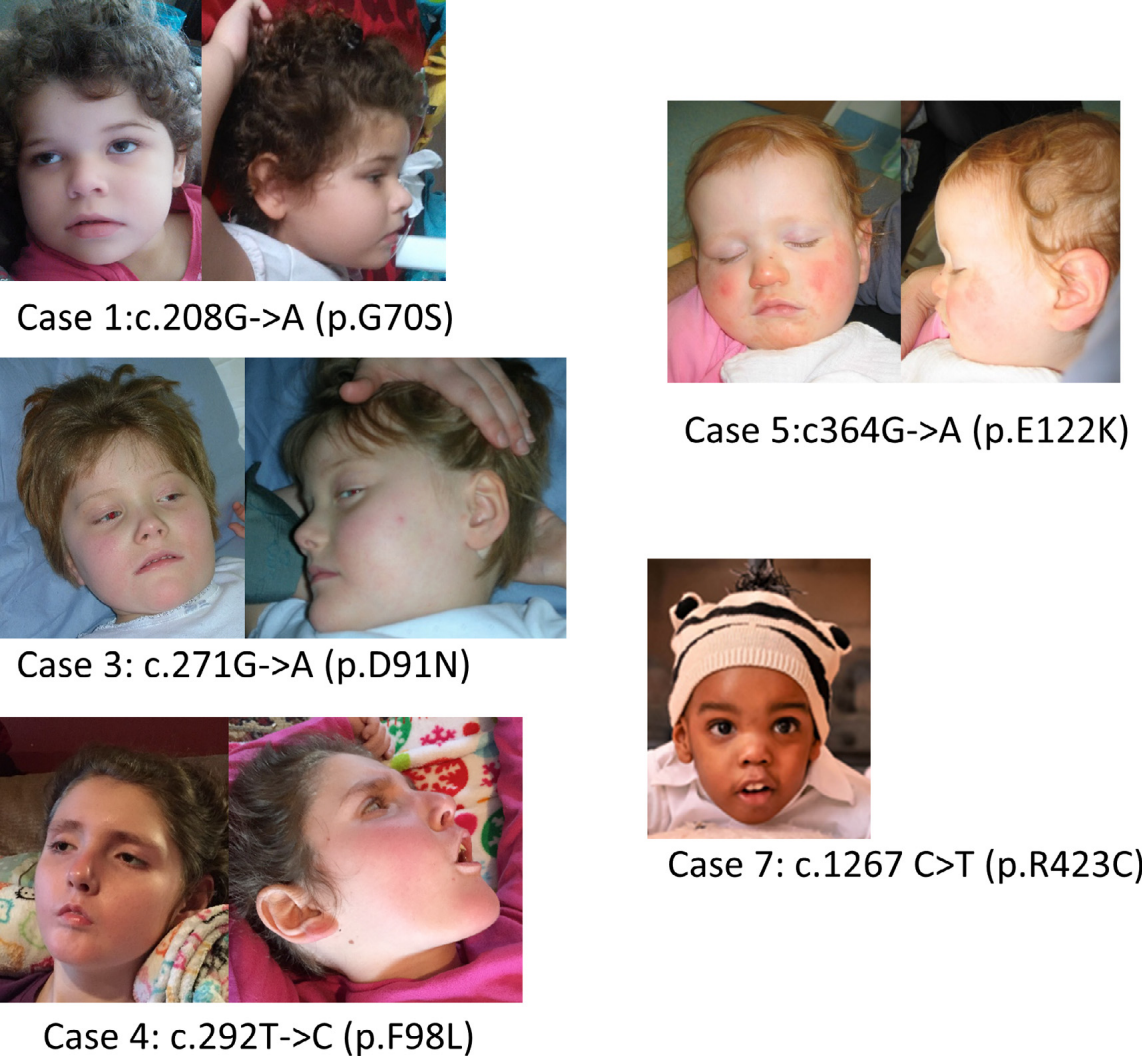
Facial dysmorphologies in five of the cases reported in this study.

Apart from case 2, we were able to obtain photographs of all the children reported here (Fig. 1). We examined these, along with reports from their clinicians, to establish whether the features reported in the two girls described by Nakajima et al. (2015) were seen consistently, to establish a possible dysmorphic phenotype for patients with *EEF1A2* mutations. The features which were common in the original two patients were deep set eyes, epicanthus, depressed nasal bridge, tented upper lips with high arch palate and an everted lower lip. Of our seven patients only three had any remarks with regards to possible dysmorphisms and there were no commonalities among the descriptions. However, on examination of the photographs it is possible to see certain distinct features emerging. Table 2 shows a summary of the facial features seen in our patients together with those described in Nakajima et al. (2015). The epicanthus and depressed nasal bridge described by Nakajima et al. (2015) are most likely due to ethnic origins, since these are not seen or described in our patients except in case 3 and case 5, respectively. A more common finding is a broad nasal bridge. The lower facial features were more consistent with 5/6 of our patients displaying a tented upper lip and eversion of their lower lip, associated with downturned corners of mouth. This is clearly demonstrated in cases 1, 3, and 5 and more subtly in cases 4 and 7 (Fig. 1). None of our patients were reported to have abnormal palates. Additional features which may be of significance are full cheeks, which have been mentioned by Nakajima et al. (2015), and large ears, but these are difficult to fully quantify given the modest numbers of cases and absence of detailed measurements.

**Table 2:** Dysmorphological facial features seen in individuals with mutations in *EEF1A2*

	Nakajima et al. (2015)		Case 1	Case 3	Case 4	Case 5	Case 6	Case 7
	Patient 1	Patient 2						
Deep set eyes	+	+	−	−	−	−	+	−
Epicanthus		+	−	+	−	−	−	−
Depressed nasal bridge	+	+	−	−	−	+	−	+
Broad nasal bridge	+	+	+	+	+	+	+	+
Tented upper lip	+	+	+	+	+	+	n.a.	+
High palate	+	+	−	−	−	−	−	−
Everted lower lip	+	+	+	+	+	+	n.a.	+
Downturned corners of mouth	+	+	+	+	+	+	n.a.	+

n.a., not able to be assessed.

### Predicted effects of the mutations on the protein

All the known mutations are missense, changing a single, highly conserved, amino acid. Table 3 lists all changes found at the DNA and protein level, together with the predicted effect of each amino acid substitution on the eEF1A2 protein.

**Table 3:** Summary of changes found in EEF1A2 by exome sequencing in individuals with epilepsy/SID

Protein change	DNA change	No. cases	PolyPhen‐2 prediction; HumDiv/HumVar score (Benign 0 to Probably Damaging 1)	SIFT prediction; Probabilities <0.05 are predicted to be deleterious	Location with respect to known binding sites, or functional data^b^
G70S^a^	G208A	3	Probably damaging; 0.998/0.980	Affect protein function; 0.00	Close to eEF1B binding site
**I71L** a	A211C	1	Possibly damaging; 0.864/0.995	Affect protein function; 0.00	Close to eEF1B binding site
**D91N** a	G271A	1	Probably damaging; 1.000/0.978	Affect protein function; 0.00	Overlaps eEF1B binding site
**F98L** a	T292C	1	Benign; 0.138/0.145	Affect protein function; 0.00	Overlaps eEF1B binding site
E122K^a^	G364A	2	Probably damaging; 1.000/0.999	Tolerated; 0.16	Affects translational fidelity in yeast; close to GTP/GDP‐binding site
**E124K** a	G370A	1	Benign; 0.101/0.072	Affect protein function; 0.03	No direct overlap with known binding sites but close to GTP/GDP‐binding site
D252H	G754C	1	Probably damaging; 0.962/0.980	Affect protein function; 0.00	Overlaps eEF1B binding site
**R423C** a	C1267T	1	Benign; 0.054/0.092	Affect protein function; 0.00	Buried

a Reported in this study, in bold = reported for the first time.

b Based upon experimental structural and/or functional data of equivalent amino acid residue in yeast eEF1A (Sandbaken and Culbertson 1988; Andersen et al. 2001; Soares et al. 2009; Crepin et al. 2014).

**Figure 2:**
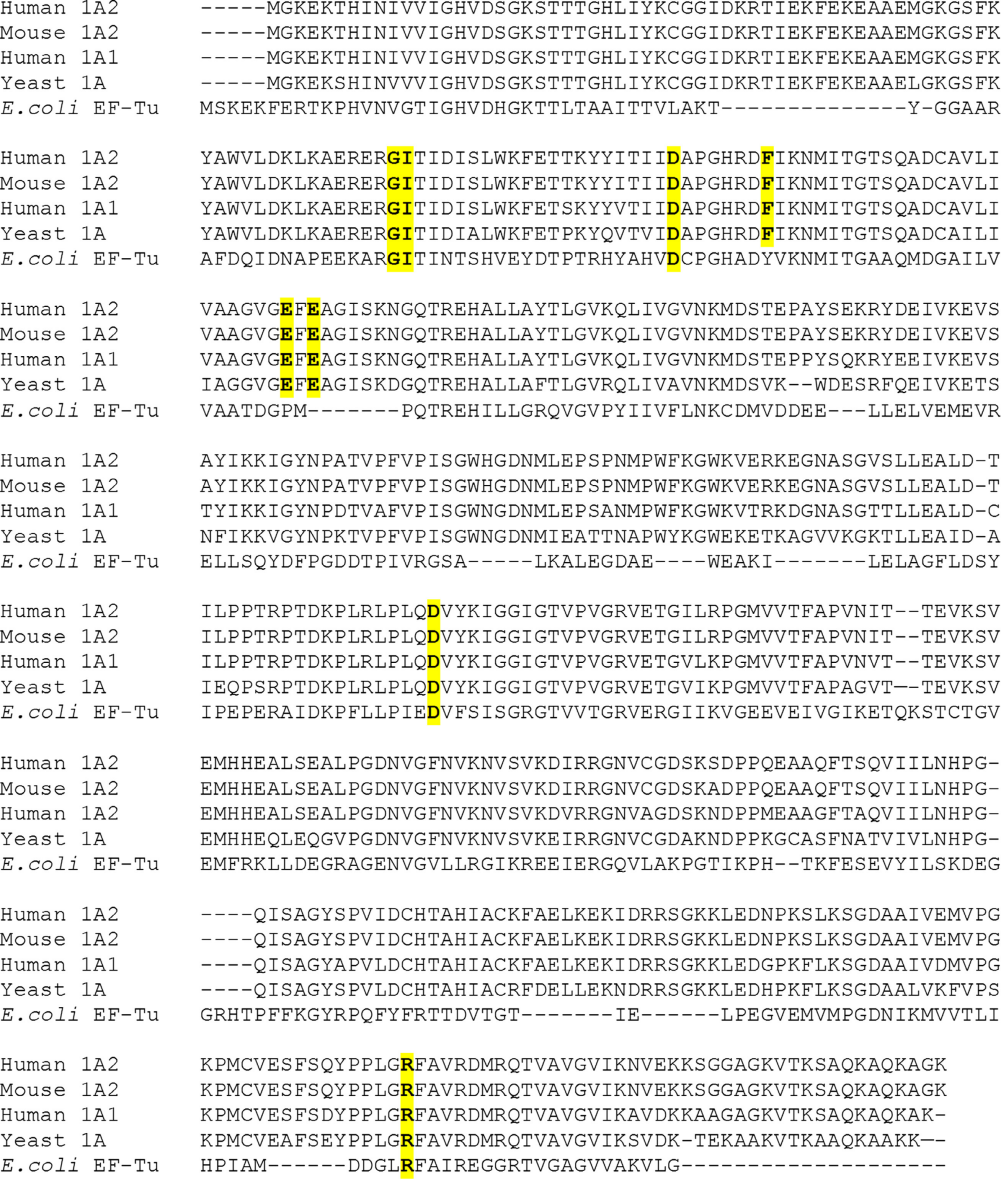
Evolutionary conservation of amino acids mutated in *EEF1A2* in individuals with epilepsy.

Each of these mutations is strikingly conserved throughout evolution. Although the specific EEF1A2 isoform seen in neurons is not found in species lower than vertebrates, genes encoding EF1alpha are seen in all life forms. If we use all isoforms for comparison, therefore, the amino acids that are mutated in five cases are strictly conserved throughout evolution to *E.coli*, and the remaining three are conserved in yeast (*S. cerevisiae*, Fig. 2). This dramatic level of conservation clearly suggests strong selective pressure and functional consequences of mutating any one of these amino acids.

## Discussion

We have identified a series of further novel de novo missense mutations in *EEF1A2* in patients with epilepsy, and thus build on the recently expanding evidence implicating *EEF1A2* as a key player in a subset of related neurological syndromes.

None of the mutations is seen in the Exome Aggregation Consortium (ExAc) database that contains data from over 60,000 individuals. Indeed, *EEF1A2* has been identified as one of the top thousand genes in the human genome considered to be subject to excessive constraint (Samocha et al. 2014); there are no known coding polymorphisms. It is also noteworthy that a spice site mutation in *EEF1B2* that encodes eEF1B*α*, the GTP exchange factor for eEF1A, was detected in a consanguineous family with nonsyndromic intellectual disability (Najmabadi et al. 2011), highlighting the importance of this pathway for cognitive and neurological disorders.

All the mutations we identified are likely to impact on the structure or function of the protein according to either PolyPhen, SIFT analysis, or both. With the exception of R423C, all mutations in eEF1A2 are solvent accessible and located at or adjacent to known binding sites from yeast or rabbit structural or mutagenesis data. The R423C mutation is buried at the interdomain junction of the protein and is thus likely to have consequences for the protein structure or conformation.

The two cases with E122K mutations may suggest a possible mechanism by which mutations in *EEF1A2* cause neuronal dysfunction, as the equivalent mutation in yeast was serendipitously characterized many years ago, and found to result in translational infidelity (Sandbaken and Culbertson 1988). The two children with E122K mutations both have an ataxia gait; ataxia has also been seen in individuals with mutations in genes encoding other proteins that are involved in the control of translational fidelity, including a missense mutation in eEF2 that underlies spinocerebellar ataxia SCA26 (Hekman et al. 2012). In addition, a mutation in an editing‐defective form of alanyl tRNA synthetase causes ataxia and neurodegeneration in mouse (Lee et al. 2006), and mutations in the gene encoding glutamine tRNA synthetase cause microcephaly and intractable epilepsy (Zhang et al. 2014). Whether similar molecular defects are seen in neurons of children with other mutations in *EEF1A2* is as yet unknown.

The newly discovered E124K mutation is of particular interest. With the caveat that it is seen in a single case (case 6) it clearly has the least severe clinical effect, with the child concerned having coped in mainstream school (albeit with moderate ID) until recently. This site is highly conserved, but does not appear to overlap completely with known binding sites for eEF1B or GTP (Soares et al. 2009). Children with less severe ID may be under represented in research cohorts used for exome sequencing analysis; in this case, the presentation of epilepsy in the neonatal period, which resolved only to remerge later in childhood, was one of the reasons for exome sequencing having been performed. It is possible, therefore, that there are other mutations in *EEF1A2* underlying many more cases of mild ID. With only a single case, however, it is impossible to know whether the less severe phenotype relates to the precise mutation, or whether there is underlying mosaicism. Indeed, this caveat applies to all cases described so far, as all have arisen de novo. Until more individuals with repeats of the same mutation have been found, this issue cannot be resolved.

The original case described with a mutation in *EEF1A2* was said to have “no evident facial dysmorphic features,” calling into question whether the characteristic features suggested by Nakajima et al. (2015) to be part of a new “*EEF1A2* syndrome” would necessarily apply in all cases, or be mutation dependent, or even a chance finding. Certainly of our seven patients, in only three cases were there any dysmorphisms noted. Following a careful review of all the photographs in cases for which we could obtain images, we conclude that indeed, there are subtle but characteristic features in common between all the individuals except case 6 (the child with the otherwise mild phenotype). The features seen in the cases we describe are consistent with those described by Nakajima et al., and include a broad nasal bridge, a tented upper lip and an everted lower lip associated with downturned corners of the mouth. It would be interesting to establish in humans the timing of the switch between eEF1A1 and eEF1A2 that occurs at ~3 weeks in rodents, and relate this to craniofacial development.

Autism has been reported in some individuals but not others. Until more cases are discovered, so that we can study more individuals with a specific given mutation, it will be impossible to judge whether a finding of autism is mutation dependent, or whether in fact individual stochastic differences are more significant than which amino acid has been mutated. Furthermore, patients with more severe intellectual disability may not meet the criteria for a diagnosis of autism.

The key question that remains is whether these mutations represent loss of function, or gain of function/dominant negative. No nonsense mutations or deletions have ever been detected in humans. While this could be because they are incompatible with life, the fact that mice with a heterozygous null mutation survive normally with no apparent physiological defects (Griffiths et al. 2012) might suggest that loss of function mutations in humans have not been detected because they do not give rise to overt disorders. However, the lack of polymorphisms in *EEF1A2* would argue against this explanation, so perhaps loss of function is simply better tolerated in mice, at least in terms of seizures and muscle function (since grip strength in heterozygous null mice is unaffected right up to 21 months, the oldest age of testing). This might suggest that the missense mutations in humans cause a gain of function, or dominant negative effect – further cases, or study of the mutations in animal models, will be needed to resolve these issues.

## Conflict of Interest

None declared.

## References

[mgg3219-bib-0001] Andersen, G. R. , L. Valente , L. Pedersen , T. G. Kinzy , and J. Nyborg . 2001 Crystal structures of nucleotide exchange intermediates in the eEF1A‐eEF1Balpha complex. Nat. Struct. Biol. 8:531–534. 11373622 10.1038/88598

[mgg3219-bib-0002] Chambers, D. M. , J. Peters , and C. M. Abbott . 1998 The lethal mutation of the mouse wasted (wst) is a deletion that abolishes expression of a tissue‐specific isoform of translation elongation factor 1alpha, encoded by the Eef1a2 gene. Proc Natl Acad Sci U S A 95:4463–4468. 9539760 10.1073/pnas.95.8.4463 PMC22512

[mgg3219-bib-0003] Condeelis, J. 1995 Elongation factor 1 alpha, translation and the cytoskeleton. Trends Biochem. Sci. 20:169–170. 7610475 10.1016/s0968-0004(00)88998-7

[mgg3219-bib-0004] Crepin, T. , V. F. Shalak , A. D. Yaremchuk , D. O. Vlasenko , A. McCarthy , B. S. Negrutskii , et al. 2014 Mammalian translation elongation factor eEF1A2: X‐ray structure and new features of GDP/GTP exchange mechanism in higher eukaryotes. Nucleic Acids Res. 42:12939–12948. 25326326 10.1093/nar/gku974 PMC4227793

[mgg3219-bib-0005] Deciphering Developmental Disorders S . 2015 Large‐scale discovery of novel genetic causes of developmental disorders. Nature 519:223–228. 25533962 10.1038/nature14135 PMC5955210

[mgg3219-bib-0006] Griffiths, L. A. , J. Doig , A. M. Churchhouse , F. C. Davies , C. E. Squires , H. J. Newbery , et al. 2012 Haploinsufficiency for translation elongation factor eEF1A2 in aged mouse muscle and neurons is compatible with normal function. PLoS One 7:e41917. 22848658 10.1371/journal.pone.0041917 PMC3405021

[mgg3219-bib-0007] Hekman, K. E. , G. Y. Yu , C. D. Brown , H. Zhu , X. Du , K. Gervin , et al. 2012 A conserved eEF2 coding variant in SCA26 leads to loss of translational fidelity and increased susceptibility to proteostatic insult. Hum. Mol. Genet. 21:5472–5483. 23001565 10.1093/hmg/dds392 PMC3516132

[mgg3219-bib-0008] Inui, T. , S. Kobayashi , Y. Ashikari , R. Sato , W. Endo , M. Uematsu , et al. 2015 Two cases of early‐onset myoclonic seizures with continuous parietal delta activity caused by EEF1A2 mutations. Brain Dev. pii: S0387‐7604(15)00233‐8. doi: 10.1016/j.braindev.2015.11.003. [Epub ahead of print]. 10.1016/j.braindev.2015.11.003 26682508

[mgg3219-bib-0009] Khalyfa, A. , B. M. Carlson , E. I. Dedkov , and E. Wang . 2003 Changes in protein levels of elongation factors, eEF1A‐1 and eEF1A‐2/S1, in long‐term denervated rat muscle. Restor. Neurol. Neurosci. 21:47–53. 12808202

[mgg3219-bib-0010] Lee, J. W. , K. Beebe , L. A. Nangle , J. Jang , C. M. Longo‐Guess , S. A. Cook , et al. 2006 Editing‐defective tRNA synthetase causes protein misfolding and neurodegeneration. Nature 443:50–55. 16906134 10.1038/nature05096

[mgg3219-bib-0011] de Ligt, J. , M. H. Willemsen , B. W. van Bon , T. Kleefstra , H. G. Yntema , T. Kroes , et al. 2012 Diagnostic exome sequencing in persons with severe intellectual disability. N. Engl. J. Med. 367:1921–1929. 23033978 10.1056/NEJMoa1206524

[mgg3219-bib-0012] Najmabadi, H. , H. Hu , M. Garshasbi , T. Zemojtel , S. S. Abedini , W. Chen , et al. 2011 Deep sequencing reveals 50 novel genes for recessive cognitive disorders. Nature 478:57–63. 21937992 10.1038/nature10423

[mgg3219-bib-0013] Nakajima, J. , N. Okamoto , J. Tohyama , M. Kato , H. Arai , O. Funahashi , et al. 2015 De novo EEF1A2 mutations in patients with characteristic facial features, intellectual disability, autistic behaviors and epilepsy. Clin. Genet. 87:356–361. 24697219 10.1111/cge.12394

[mgg3219-bib-0014] Samocha, K. E. , E. B. Robinson , S. J. Sanders , C. Stevens , A. Sabo , L. M. McGrath , et al. 2014 A framework for the interpretation of de novo mutation in human disease. Nat. Genet. 46:944–950. 25086666 10.1038/ng.3050 PMC4222185

[mgg3219-bib-0015] Sandbaken, M. G. , and M. R. Culbertson . 1988 Mutations in elongation factor EF‐1 alpha affect the frequency of frameshifting and amino acid misincorporation in *Saccharomyces cerevisiae* . Genetics 120:923–934. 3066688 10.1093/genetics/120.4.923 PMC1203584

[mgg3219-bib-0016] Soares, D. C. , P. N. Barlow , H. J. Newbery , D. J. Porteous , and C. M. Abbott . 2009 Structural models of human eEF1A1 and eEF1A2 reveal two distinct surface clusters of sequence variation and potential differences in phosphorylation. PLoS One 4:e6315. 19636410 10.1371/journal.pone.0006315 PMC2712093

[mgg3219-bib-0017] Veeramah, K. R. , L. Johnstone , T. M. Karafet , D. Wolf , R. Sprissler , J. Salogiannis , et al. 2013 Exome sequencing reveals new causal mutations in children with epileptic encephalopathies. Epilepsia 54:1270–1281. 23647072 10.1111/epi.12201 PMC3700577

[mgg3219-bib-0018] Wright, C. F. , T. W. Fitzgerald , W. D. Jones , S. Clayton , J. F. McRae , M. van Kogelenberg , et al. 2015 Genetic diagnosis of developmental disorders in the DDD study: a scalable analysis of genome‐wide research data. Lancet 385:1305–1314. 25529582 10.1016/S0140-6736(14)61705-0 PMC4392068

[mgg3219-bib-0019] Yang, Y. , D. M. Muzny , F. Xia , Z. Niu , R. Person , Y. Ding , et al. 2014 Molecular findings among patients referred for clinical whole‐exome sequencing. JAMA 312:1870–1879. 25326635 10.1001/jama.2014.14601 PMC4326249

[mgg3219-bib-0020] Zhang, X. , J. Ling , G. Barcia , L. Jing , J. Wu , B. J. Barry , et al. 2014 Mutations in QARS, encoding glutaminyl‐tRNA synthetase, cause progressive microcephaly, cerebral‐cerebellar atrophy, and intractable seizures. Am. J. Hum. Genet. 94:547–558. 24656866 10.1016/j.ajhg.2014.03.003 PMC3980424

